# Implementation of a Defecation Posture Modification Device: Impact on Bowel Movement Patterns in Healthy Subjects

**DOI:** 10.1097/MCG.0000000000001143

**Published:** 2019-02-11

**Authors:** Rohan M. Modi, Alice Hinton, Daniel Pinkhas, Royce Groce, Marty M. Meyer, Gokulakrishnan Balasubramanian, Edward Levine, Peter P. Stanich

**Affiliations:** * Department of Internal Medicine; ‡ Division of Gastroenterology, Hepatology and Nutrition, The Ohio State University Medical Center; † Division of Biostatistics, College of Public Health, The Ohio State University, Columbus, OH

**Keywords:** defecation postural modification device (DPMD), bowel duration, straining patterns, bowel emptiness

## Abstract

**Goals:**

The goal of this study was to evaluate the influence of defecation postural modification devices (DPMDs) on normal bowel patterns.

**Background:**

The introduction of DPMDs has brought increased awareness to bowel habits in western populations.

**Materials and Methods:**

A prospective crossover study of volunteers was performed that included real-time collection of data regarding bowel movements (BMs) for 4 weeks (first 2 wk without DPMD and subsequent 2 wk with DPMD). Primary outcomes of interest included BM duration, straining, and bowel emptiness with and without DPMD use.

**Results:**

In total, 52 participants (mean age, 29 y and 40.1% female) were recruited for this study. At baseline 15 subjects (28.8%) reported incomplete emptying, 23 subjects (44.2%) had increased straining, and 29 subjects (55.8%) noticed blood on their toilet paper in the past year. A total of 1119 BMs were recorded (735 without DPMD and 384 with DPMD). Utilizing the DPMD resulted in increased bowel emptiness (odds ratio, 3.64; 95% confidence interval (CI), 2.78-4.77) and reduced straining patterns (odds ratio, 0.23; 95% CI, 0.18-0.30). Moreover, without the DPMD, participants had an increase in BM duration (fold increase, 1.25; 95% CI, 1.17-1.33).

**Conclusions:**

DPMDs positively influenced BM duration, straining patterns, and complete evacuation of bowels in this study.

Defecation in its simplest form consists of 3 components: spontaneous rectal contraction (autonomic), straightening anorectal angle due to relaxation of puborectalis and external anal sphincter (largely somatic), and straining (somatic).^1^ Straining is the aspect of defecation over which individuals have the largest control from an anatomic standpoint. Many patients who struggle with chronic constipation may have abnormal rectoanal coordination with paradoxical contraction instead of relaxation of the pelvic floor during defecation, also known as dyssynergic defecation.^2^,^3^ While squatting during bowel movements (BMs) is commonly practiced in the Middle East, Africa, and Asia; western populations have transitioned to using the toilet.^4^ Recently, there has been increasing social awareness of suboptimal bowel habits including increased strain, inadequate bowel emptying, and increased time with defecation in western populations that may be related to positioning during defecation. The introduction of defecation postural modification devices (DPMDs) was developed to replicate the alignment achieved with squatting while using a toilet (Fig. 1). Prior studies addressing this topic are limited by sample size and none of them were completed with a US population. The aim of our study was to investigate the effects of DPMDs on a large sample of asymptomatic volunteers.

**FIGURE 1:**
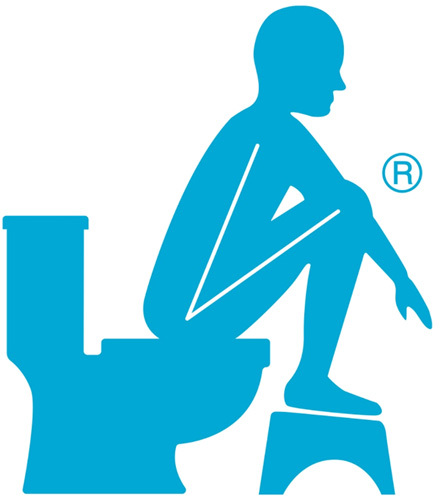
Defecation postural modification device demonstrating the change in anorectal angle. Copyright (Squatty Potty, LLC), (St. George, UT). All permission requests for this image should be made to the copyright holder.

## MATERIALS AND METHODS

The Ohio State University Institutional Review Board approved this study. Our cohort included healthy volunteers randomly selected from a total of 147 Internal Medicine residents as well as their significant others and/or spouses who would have access to the device. Individuals were expected to be able to fill out real-time survey questionnaires after each BM. Exclusion criteria included individuals with the following concerns: (1) previously used a DPMD; (2) wheelchair bound; (3) presence of ileostomy/colostomy; (4) history of small bowel resection >12 cm; or (5) currently pregnant. All surveys for this study were created using Google Form and could be downloaded directly onto a smartphone for participant convenience. Participants filled out a pre-DPMD survey anonymously that included a compilation of validated questionnaires from Bowel Disease Questionnaire,^5^ Bowel Habits in Young Adults Not Seeking Health Care,^6^ Female Bowel Function.^7^ Demographic information (sex, age, race), comorbidities (heart disease, diabetes, thyroid disease, inflammatory bowel disease, lupus, fibromyalgia, multiple sclerosis, cancer) or previous abdominal surgeries were ascertained from the initial survey. More importantly, specific information regarding bowel habits including frequency (every other day, once a daily, 2+ times per day), consistency (soft, normal, or hard), increased straining, and incomplete emptiness was obtained for each individual.

The DPMDs used in the study were the Squatty Potty (Squatty Potty, LLC, St. George, UT). Participants were instructed to use the devices according to manufacturer recommendations.

At study initiation, participants recorded BMs for a total of 4 weeks with the first 2 weeks without DPMD and subsequent 2 weeks with DPMD. BMs were recorded on Google Form with participants asked to respond to the same 5 questions: (1) participant number; (2) DPMD usage; (3) straining patterns; (4) bowel emptiness; and (5) time of BM (Table 1). Both straining and bowel emptiness scales are further delineated in Table 1. Responses regarding each BM were automatically uploaded and could be filtered based on participant number and DPMD usage. Participants were sent a postsurvey at the end of the study with subjective questions on their experience with the device.

**TABLE 1:**
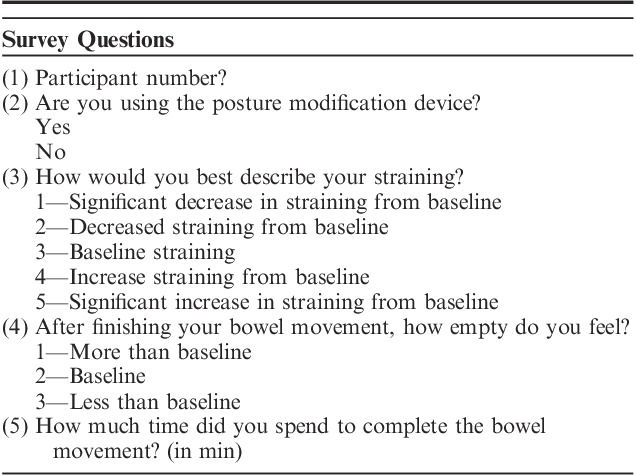
Bowel Movement Survey

### Statistical Analyses

Categorical variables were summarized with frequencies and percentages while the continuous variable age was summarized with its mean and SD. A mixed model for log-transformed duration was fit and mixed proportional odds ordinal logistic regression models were fit for bowel emptiness and straining. All models contained random subject intercepts and fixed effects for use of the DPMD. It is important to note that the analysis is intrasubject as it looks at changes in the participant’s outcomes before and after DPMD use. Results for duration are reported as fold increases when the DPMD was not used as compared with when it was. Results for bowel emptiness and straining are reported as odds of more emptiness and less straining while using the DPMD as compared to without. The 3 above models were refit separately among the subsets of participants who reported incomplete emptying, increased straining, and blood on toilet paper at baseline. The χ^2^ tests were used to compare the characteristics of participants who plan to continue to use their DPMD with those who do not plan to continue use. All analyses were completed with SAS 9.4 (SAS Institute, Cary, NC).

## RESULTS

### Preintervention Survey

In total, 52 participants (mean age, 29 y and 40.1% female) took part in this study. At baseline, 15 subjects (28.8%) reported incomplete emptying, 23 subjects (44.2%) had increased straining with defecation, and 29 subjects (55.8%) noticed blood on their toilet paper in the past year (Table 2).

**TABLE 2:**
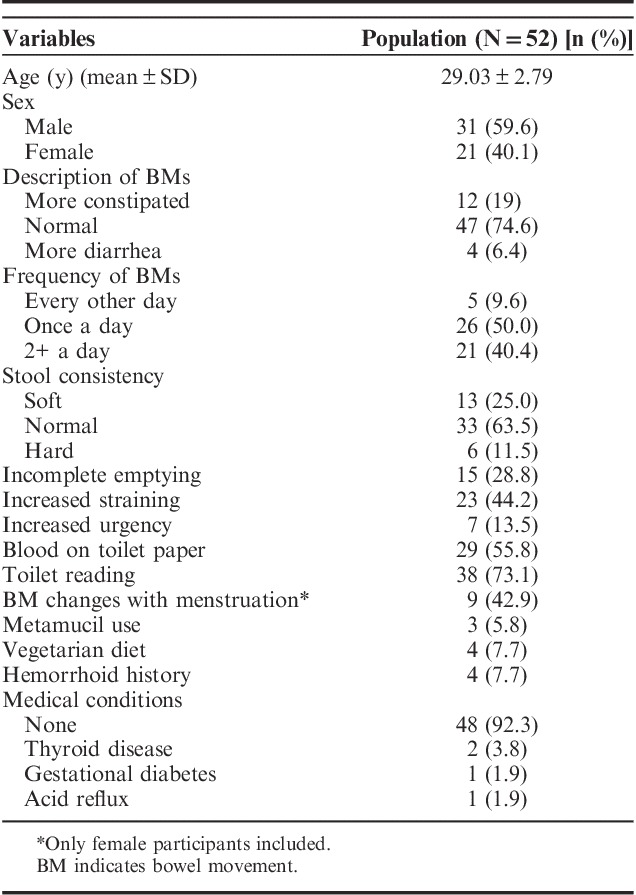
Participant Characteristics From Presurvey Before Using the Defecation Postural Modification Device

### Effect of DPMD on Primary Outcomes

**FIGURE 2:**
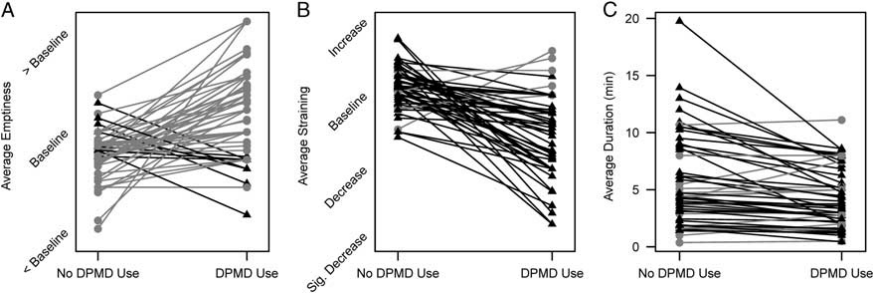
Individual trends for emptiness (A), straining (B), and duration (C). Positive slopes are shown in gray with circles and those with a negative slope are black with triangles. DPMD indicates defecation postural modification device.

A total of 1119 BMs were recorded (735 without DPMD and 384 with DPMD). Utilizing the DPMD resulted in increased bowel emptiness [odds ratio (OR), 3.64; 95% confidence interval (CI), 2.78-4.77] and reduced straining patterns (OR, 0.23; 95% CI, 0.18-0.30) (Table 3A). When not using the DPMD, participants also had an increase in BM duration (fold increase, 1.25; 95% CI, 1.17-1.33) (Table 3B). The model was refit separately among subsets of participants who reported incomplete emptying, increased straining, and blood on toilet paper at baseline and again showed DPMDs positively influenced results (Table 3). All 3 outcome measures were subsequently graphed based on individual participants and again showed increased bowel emptiness (44/52; 85%), reduced straining (47/52; 90%), and reduced duration (37/52; 71%) with DPMDs (Figs. 2A–C).

**TABLE 3:**
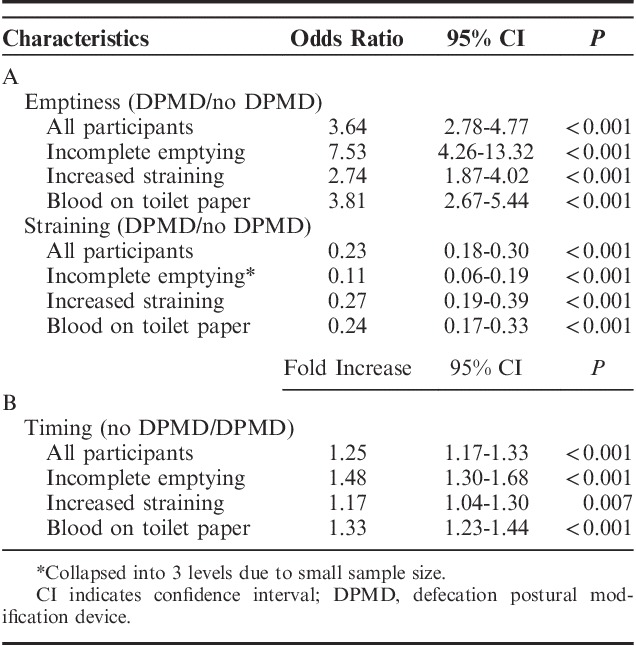
Assessing the Impact of the DPMD on Emptiness/Straining (A) and Timing (B) Stratified by Participant Characteristics

To evaluate if any specific subgroup of subjects had a better response to intervention, 5 variables were added to the model separately along with an interaction between the variable and DPMD use. The 5 variables included sex (male vs. female), more constipated (vs. normal/diarrhea), hard consistency (vs. normal/soft), increased straining (vs. no straining), and decreased emptiness (vs. normal emptiness). For the outcome of emptiness, DPMD worked better for males (OR, 4.91 vs. 1.97; *P*=0.001), those with constipation (OR, 8.98 vs. 3.10; *P*=0.002), and those who reported decreased emptiness (OR, 7.96 vs. 2.84; *P*=0.001). DPMD improved straining outcomes for participants who reported constipation (OR, 0.08 vs. 0.28; *P*<0.001) and participants who reported decreased emptiness (OR, 0.11 vs. 0.29; *P*=0.001). Finally, DPMD reduced timing for males as compared with females (1.32 vs. 1.10 fold increase; *P*=0.010) and worked better for those who reported decreased emptiness (1.48 vs. 1.17 fold increase; *P*=0.003).

### Postintervention Survey

The poststudy survey indicated that 35 patients (67.3%) plan to continue to use their DPMD (Table 4). Compared with data trends depicted in Figures 2A–C, participants had less notable differences when asked if there was an improvement in emptiness (85% vs. 50%), straining (90% vs. 65%), or duration (71% vs. 50%), respectively. Multivariate analysis indicated incomplete emptying was the only variable associated with continuation of DPMD after study conclusion.

**TABLE 4:**
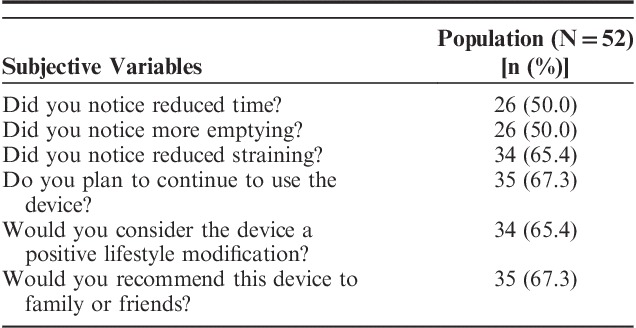
Defecation Postural Modification Device Postsurvey Responses

## DISCUSSION

There has been a significant interest in and usage of DPMDs in popular culture, but there is a lack of research into their effectiveness in a US population. Our results indicate that DPMDs positively influenced defecatory time, straining, and complete evacuation of bowels in a presumed healthy population.

Rectal emptying during squatting is facilitated by anorectal angle straightening, resulting in higher rectal pressure and lower anal pressures with possible levator ani relaxation.^8^ Previous studies have documented that squatting improved the angle of the anorectal canal, reduced strain, increased sensation of adequate bowel emptying, and decreased time associated with defecation when compared with sitting.^1^,^4^,^9^ Our study suggests that by emulating characteristics of squatting, DPMDs can provide similar benefits to patients.

Although it has been shown that straining patterns may be related to pathologic conditions,^10^ we felt it was important to understand the influence it has on a healthy population. A 2017 study confirmed the “3 and 3” metric for frequency of BMs (3 BMs/d to 3 BMs/wk), while also suggesting that men (3 to 5) and women (2 to 6) have varying Bristol stool form scale values.^11^ We were surprised to find that over half of our participants had found blood on their toilet paper in the past year. Moreover, a quarter of our so-called “healthy population” reported experiencing incomplete emptying and approximately half indicated increased straining. It is important to consider that our results may be confounded by the fact that certain participants carry gastroenterology diseases such as irritable bowel disease, functional constipation, or defecatory disorder that is further complicated by the stressors and hours of a medical residency. Given the findings of our study, future studies may want to revisit the notion of healthy BM in the average American.

There is substantial literature on chronic constipation and irritable bowel syndrome type C; however, there are minimal data on normal bowel patterns. One study assessed normal bowel habits for 425 women and found that the average time for defecation was 6.1±4.7 minutes for patients within age 18 to 35 years old.^7^ In addition, one third of all participants included in the aforementioned study read on the toilet with the majority of them trying to relax or distract themselves. Interestingly, a recent Iranian study found that more than half of survey patients (52.7%) were toilet readers with significantly longer time on the toilets.^12^ Toilet readers also seemed to have an increase prevalence of hemorrhoids; however, this was not statistically significant. It is important to note that our study showed similar results in terms of both timing (5.60 min without DPMD vs. 4.24 min with DPMD) and percentage of toilet readers (73.1%, 38/52 participants).

DPMDs may offer a nonpharmacologic option for a common diagnosis such as constipation. The burden of chronic constipation has increased from 4 million ambulatory visits annually between 1993-1996 period to 7.95 million visits during the 2001-2004 timeframe.^13^ Moreover, recent review articles suggest that between 37% and 40% of patients who suffer from chronic constipation are diagnosed with dyssynergic defecation.^2^,^3^ Although biofeedback therapy has been shown to improve symptoms in this population, this treatment modality is not always readily available or financially possible for our patients. This study demonstrates that DPMDs may offer a low-cost noninvasive option that can be considered in both the outpatient and hospital setting. There is a potential cost-saving benefit with this device that may reduce the frequency of health care visits and medication expenditures for constipation.

There are important limitations of this study to address. First, both straining and emptiness were measured on a subjective scale due to study design. It is also significant that there was approximately half the number of BMs recorded using the DPMD versus no DPMD. The likely explanation is related to the participant population having limited access to their DPMD during work hours. In addition, participant bias may have been present when recording BMs without seeing a true clinical benefit, as there were discrepancies between the recorded data trends and postintervention survey. Finally, using resident physicians as our study population may limit the applicability of our results to patient populations who are presumed to be less healthy. Future studies can address these limitations by considering utilization of high-resolution anorectal manometry and balloon expulsion time to investigate defecatory dynamics in the sitting and squatting positions.^14^ Moreover, focusing on specific pathology including hemorrhoids or opioid-induced constipation may be worthwhile to explore.

In conclusion, DPMDs positively influenced BM duration, straining patterns, and complete evacuation of bowels in this study of healthy volunteers. This is the first study assessing the role of DPMDs in the United States. DPMDs offer a nonpharmacologic option for those individuals who suffer from inadequate bowel emptying or increased straining.
